# The Aesthetics of Encounter: A Relational-Performative Design Approach to Human-Robot Interaction

**DOI:** 10.3389/frobt.2020.577900

**Published:** 2021-03-16

**Authors:** Petra Gemeinboeck

**Affiliations:** ^1^ Department of Media Theory, University of Applied Arts Vienna, Vienna, Austria; ^2^ Centre for Transformative Media Technologies, School of Arts, Social Sciences and Humanities, Swinburne University of Technology, Melbourne, VIC, Australia

**Keywords:** human-robot interaction design, aesthetics, performativity, agency, design, movement

## Abstract

This article lays out the framework for relational-performative aesthetics in human-robot interaction, comprising a theoretical lens and design approach for critical practice-based inquiries into embodied meaning-making in human-robot interaction. I explore the centrality of aesthetics as a practice of embodied meaning-making by drawing on my arts-led, performance-based approach to human-robot encounters, as well as other artistic practices. Understanding social agency and meaning as being enacted through the situated dynamics of the interaction, I bring into focus a process of *bodying-thinging;* entangling and transforming subjects and objects in the encounter and rendering elastic boundaries in-between. Rather than serving to make the strange look more familiar, aesthetics here is about rendering the differences between humans and robots more relational. My notion of a relational-performative design approach—*designing with bodying-thinging—*proposes that we engage with human-robot encounters from the earliest stages of the robot design. This is where we begin to manifest boundaries that shape meaning-making and the potential for emergence, transformation, and connections arising from intra-bodily resonances (*bodying-thinging*). I argue that this relational-performative approach opens up new possibilities for how we design robots and how they socially participate in the encounter.

## Introduction

Social robots are designed to operate, mediate, or directly engage in social scenarios, provoking the question of how a machine becomes a social participant in these encounters. “Good” interaction, it is often assumed, should feel “natural,” i.e., reminiscent of social interaction between humans (Hegel, 2012; Dautenhahn, 2013; Lindblom and Alenljung, 2020). But what if “there is no such thing as ‘natural interaction’” (Dautenhahn, 2013)? Human social interaction is a complex dynamic phenomenon, embedded in a specific cultural setting, shaped by environmental and social contexts, and reflective of lived experiences and relationships. Given robots’ vastly different mechanical and cognitive makeup and, not least, complete lack of “lived experience” (Dewey, 1934), the notion of “natural” in the context of robotic artifacts is even more problematic. Mindell thus argues that it is the designer’s beliefs and intentions that shape a robot’s “abilities and its relationships with the people who use it” (2015: 10). In other words, a robot’s social competences are not “natural” but shaped by the designer’s beliefs of what “natural interaction” looks like. The challenge of human-machine communication, according to Suchman, lies in the differences and “deep asymmetries” between humans and machines “as interactional partners” (2007: 11). Yet “obscur[ing] enduring asymmetries,” e.g., through humanlike features or behaviors, does not resolve them and “people inevitably rediscover those differences in practice” (Suchman, 2007a: 13). While modeling human-robot relationships after human relationships (Jones, 2018; see also Castañeda and Suchman, 2014; Hegel, 2012; Dautenhahn, 2013) may ensure a certain familiarity in our encounters with robots, mimicking our social relationships with these new social entities can also only be just that: a figment of our human imagination curbed by what we already know—or assume to know—about the human and being social.

This article introduces an alternative, arts-led approach to imagining our relationships with robots that embraces their difference and aims to take on a mediating role between human interactors and these potentially new social entities by locating itself in the middle of the encounter. My Machine Movement Lab (MML) project opens up an intimate link to performance-based inquiries into the relational enactment of human-robot encounters to investigate the generative potential of movement and its dynamic qualities to enact meaning with abstract robotic artifacts. Starting in 2015, the practice-based research project set out to explore the possibility of meaningful encounters with robots in ways that playfully employ machines’ unique otherness. To situate^1^ such strange artifacts in our social environment, we explore the potential of qualitative movement dynamics, “com[ing] to the fore in dance” (Sheets-Johnstone, 2012: 49), that afford them a relational quality that is unique to their machinic embodiment. Importantly, this relationality is not a capacity built-into the robot but rather is generated and comes to effect in the interactional process of each particular encounter. As a designer, positioning oneself in the middle of the encounter and the relationships it produces deliberately undermines focusing on the individualism of interacting agents and, instead, promotes attending to the crisscross of perceptual flows, movement dynamics, and emergent effects that give rise to meaning. This is a fundamentally aesthetic process as I will attempt to lay out in this article, rendering aesthetics core to meaning-making in human-robot interaction.

My notion of aesthetics is not confined to the purview of philosophy and art theory, where “aesthetics experience” is evoked or sustained by an “aesthetics object” and delineated as the appreciation of beauty in all its forms (Osborne, 1986). Cultural theory and other disciplines, in recent years, have worked to realign our understanding of aesthetics with its Greek origins *aisthēsis* (perception), often described as an experience of sensorial or sensate binding, “a connectivity based on the senses” (Bal, 2015: 152; see also Bennett, 2012). My understanding of aesthetics pertains to how we make meaning, arising from particular relations, and patterns thereof, that resonate with us, predicated upon our bodily sense-making of the world. Aesthetics here is a mode of embodied and distributed meaning making (see Johnson, 2018; Johnson, 2007; Lindblom, 2020; Lindblom, 2015), tightly linked to a relational understanding of agency, enacted through the very same relational dynamics (see Barad, 2007; Suchman, 2007a). Bringing into dialogue Dewey’s pragmatist aesthetics (1934) and central concepts from embodied cognition, Johnson argues that “all meaningful experience is esthetic experience” (2018: 2). It draws on all the processes by which we make sense of the world and “enact meaning through perception, bodily movement, feeling, and imagination” (Johnson, 2018: 2; see also Alexander, 2013). Meaning is thus fundamentally “relational, experiential, and enactive” (Johnson, 2018: 244), framed in a particular social, material, cultural, and historical context. Looking at meaning-making in human-robot interaction, my proposed esthetic account also draws on a performative^2^ understanding of agency, where agency is not a property, held by an individual agent, but rather is “a matter of intra-acting … an enactment” (Barad, 2007: 178). The perception of agents or someone/something being perceived as agential then is an effect of agential enactment and inherently relational. Speaking to our representational and differential practices and how they may shape our relationship with robots, the performative is an important dimension in our meaning-making processes, both as part of the design process and interactional experience (see *Bodying‐Thinging*).

In this article, I will attempt to lay out the framework for *relational-performative aesthetics in human-robot interaction*, drawing on performative new materialist accounts (e.g., Barad, 2007; Suchman, 2007a; Gamble et al., 2019) and embodied, distributed meaning-making (e.g., Johnson, 2007; Johnson, 2018; Lindblom, 2020; Lindblom, 2015), and grounded in my Machine Movement Lab (MML) project^3^ in conversation with other artistic approaches (Demers 2016; Penny, 2016). Aesthetics here is a site of research and both a theoretical lens and a material practice of inquiry into performative, relational meaning-making in human-robot interaction. I begin by discussing positions and practices in and around human-robot interaction research that are relevant to laying out my argument (*Relevant Positions and Practices*). Following this, I introduce my MML project and its core method of Performative Body Mapping (PBM) that harnesses dancers’ tactile-kinaesthetic expertize to explore the social potential of human-robot relationships as *difference in relation* (*Difference in Relation: Machine Movement Lab*) and how it unhinges and makes elastic subject-object boundaries through a process of *bodying-thinging* (*Bodying-Thinging*). In (*Designing with Bodying-Thinging*), I argue that our meaning-making encounters do not only begin once a robot design is complete and able to partake but is put in motion as soon as we begin to imagine, experiment with, prototype, test, and *make meaning of* the artifact’s design. Finally, I briefly discuss my current research in expanded performance-making, (*Dancing with the Nonhuman*), revolving around the negotiation of different perceptual worlds facilitated through Relational Body Mapping (RBM), and complete with a (*Discussion and Summary*).

## Relevant Positions and Practices in and Around Human-Robot Interaction Research

One might argue that modeling human-robot relationships after human-human relationships has shown that this mimicking approach has succeeded in rendering robots more acceptable and easier to interact with (Hegel et al., 2009; Dautenhahn, 2013; De Graaf and Allouch, 2013), yet we can also find many studies reporting on the challenges brought forth by this assumption (Lee et al., 2016; Vlachos et al., 2016; Šabanović, 2010). One major concern is that humanlike appearance and behavior often evoke expectations of human-level cognition and empathy, which cannot only lead to frustration (Dautenhahn, 2013) but beguile vulnerable users through an illusory sense of experiencing a mutual relationship (Turkle, 2011). Critical voices from Science and Technology studies have called for “a more differentiated set of starting points for the robot” (Castañeda and Suchman, 2014: 340) that evade generic, universal assumptions about “the human” and could open up other possibilities for human-robot relations (Castañeda and Suchman, 2014). From a creative perspective, such a mimicking approach relies on what we already know—or assume to know—about social relationships and our capacity to form them, posing restrictions not only on what a robot could be but also what relationships we could have with them.

Understanding meaning-making as a fundamentally esthetic and embodied process, situated and unfolding in the interaction scenario itself, moves the design focus into the middle of possible interactional scenarios and puts the spotlight on difference in relation. This relational, performative view contrasts human-centric design approaches founded on the belief that humanlike features and familiar behaviors can be orchestrated to give social agency to a robot (Alač, 2015; Jones, 2017). Social robot design approaches and studies largely limit esthetic concerns to a robot’s physical appearance, often specifically referring to its purpose as creating visual appeal (Salvini et al., 2010; Hegel, 2012; De Graaf and Allouch 2013; Hoffman and Ju 2014; Paauwe et al., 2015). The enactment of affordances, which necessarily involves ecological and perceptual considerations that can render evaluation more challenging, is often approached as a matter of an agent’s capabilities (Paauwe et al., 2015) or behavioral functions (Hegel, 2012) that can be designed and programmed into a robot (Alač, 2011; Jones, 2018). Human-centric design then becomes a matter of technocentric problem-solving where appearance and capability are viewed as separate design components, rendering the robot a friendly-looking “physical container” (Ziemke, 2016) for social functions.

Coming back to the question of “natural interaction,” one major assumption underlying humanlike robot designs is that successful communication “is founded on what communicators already have in common” (Sandry, 2016: 179; see also Boer and Bewley, 2018). It frames social communication as a process that has “a correct outcome” or predefined protocol, where potentially ambiguous meanings or multiple interpretations would be, in Sandry’s words, “an undesirable risk that should be eliminated” (2016: 179). But, this desire for control in the interactional exchange may lead to a simplistic, problem-focused approach to social encounters, driven by what makes them “amenable to technological intervention” (Šabanović, 2010) yet blind to the emergent dynamics and effects that are core to our social, embodied interactions. From an embodied and enactive cognition perspective, social interaction “cannot be reduced to so-called ‘social information transfer’” (Lindblom, 2020: 10). Rather, social interaction is always relational (Gallagher, 2005; Di Paolo et al., 2010; Fuchs, 2016), where meaning- or sense-making emerges dynamically through “creative co-regulated socially embodied interactions” (Lindblom, 2020: 10; see also Johnson, 2018). Instead of accessing our world through representations, we bodily participate in the generation of meaning, “often engaging in transformational and not merely informational interactions; [we] enact a world” (Di Paolo et al., 2010: 39). My relational-performative esthetic approach to human-robot interaction builds on the embodied, enactive approach, aligned with a performative understanding of how agency is relationally enacted, to develop a deeper understanding of how meaning is bodily negotiated in human-robot encounters.

Much of our embodied, social meaning-making process involves movement and, in particular, movement qualities, allowing us to rhythmically coordinate with others through interaction (Di Paolo et al., 2010) and bodily resonate with affective qualities or environmental affordances (Fuchs and Koch, 2014). Looking at emotions “as embodied responses to meaningful situations” (Fuchs, 2016: 1), Fuchs and Koch understand motion and emotion as “intrinsically connected: one is *moved by movement* (perception; impression; affection) and *moved to move* (action; expression; e-motion)” (2014: 1). A number of researchers have thus explored the expressive potential of motion design beyond imitating human movement and, instead, focusing on how it can affect our interpretation of abstract, non-humanlike robotic artifacts. Levillain and Zibetti have investigated how non-humanlike “behavioral objects” open up possibilities for intuitive connection based on simple, evocative movement patterns (2017). Using the CoBot platform, Knight and Simmons have studied how expressive motion allows for a robot’s movements to be interpreted as simple mental states, e.g., happy vs. sad (2014). Jochum et al. discuss theatrical performance practices and entertainment robots, showing that strategies adopted from traditional puppetry can inform creative solutions for robot motion design (2017)^4^. LaViers et al. (2018) have explored tools and techniques from choreography and somatics that can inform the development of expressive robotic systems.

Robot design practices that place movement and its potential for social meaning-making at the center of the design process, from the very beginning, are much rarer. Drawing on techniques from abstract character animation, Hoffman and Ju argue that a “robot’s motion can clue users into what actions and interactions are possible,” thus playing a significant, yet still “widely under-recognized” (2014: 95) role in human-robot interaction. Dominated by pragmatic and visual approaches, social robot designs, if at all, commonly only integrate movement qualities later in the process, once mechanical and visual development are completed (Hoffman and Ju, 2014). In a motion-centric approach, in contrast, the robot’s design is shaped by the communicative potential of movement, unfolding in an ongoing conversation with pragmatic and appearance-related issues (Hoffman and Ju, 2014). In fact, it would be difficult to imagine how a design process oriented toward the quality of movements could not take on an iterative, integrated approach, given that a robot’s movement potential relies on its mechanical workings and its perceived effect cannot be separated from its visual presence.

An important example demonstrating this motion-centric approach is Shimon, an interactive robotic marimba player (Hoffman and Weinberg, 2011), featuring a socially expressive and communicative head and four arms that move along a shared rail. Bringing together mechanical looks and gestural movement, the head supports the robot’s interaction and improvisation with its human band members (Hoffman and Ju, 2014). Importantly, Hoffman and Ju point out that a motion-centric approach that invests in carefully designed movement qualities to develop a robot’s “complexity and sophistication” (2014: 93) can lead to more abstract, geometric designs that afford more feasible and rapid prototyping and testing than humanoid designs. Within this context, Sirkin et al. (2016) have developed a design approach, where movement does not drive new robot designs but rather turns existing objects into communicative social artifacts. Studying “objects *in motion* because interactivity implies sociability” (Sirkin et al., 2016: 95; see also Yang et al., 2015), they have developed a series of expressive robotic artifacts that expand everyday objects, including a mechanical ottoman, emotive dresser drawers, and a roving trash barrel. Aiming to bestow the artifacts with expressive personalities, Sirkin et al. involved practitioners from dance, improvisational theater, and stage theater to operate the objects employing Wizard of Oz techniques in improvisational experiments (2016).

Aesthetics, where referred to in the approaches above, is still only considered with respect to a robot’s physical form. In contrast, Popat and Palmer (2005) identify aesthetics as the common ground from which genuinely creative dialogues between performers and technologists can arise and offer an insightful account for embodied knowledge exchange between a dancer and roboticists, mediated by a six-legged robot prototype. Recent creative work in soft robotics has opened up the design space for social robots by offering provocative modes of inquiry into their appearance, movement, and interaction potential (Boer and Bewley, 2018; Jørgensen, 2019). Embracing “the robot as a quasi-other,” Boer and Bewley explore alternative ways for human-robot communication through the performative potential of abstract soft robots, based on kinetic expression (2018). Jørgensen has proposed an extensive framework for aesthetics of soft robotics that develops a dialogue between artistic practices and material, ecological thinking to explore the performative potentials and sociomaterial consequences of rendering a robot soft (2019). Both esthetic approaches focus on the interplay between the unique material affordances of soft robots and its expressive movement qualities, e.g., through softness (Jørgensen, 2019) and elasticity (Boer and Bewley, 2018). In contrast to this foregrounding of material performativity, employing movement as a medium to evoke a character or personality (e.g., see Sirkin et al., 2016; Knight and Simmons, 2014; Hoffman and Ju, 2014) suggests that it is the expression of a given character’s qualities that shapes the robots’ social potential.

Agential enactment and its social effects play an important discursive role in science and technology studies (see Suchman, 2007a; Alač, 2015; Jones, 2018) and cognitive anthropology (see Malafouris, 2013) and gain influence in human-computer interaction research (see Wright, 2011; Hylving, 2017; Frauenberger, 2019). This article argues that the transformative potential of interaction dynamics opens up rich opportunities for how we relate to robots and brings with it new pathways and challenges for how we approach human-robot interaction design. According to Kroos et al. (2012), the question of agency within the context of robots “seems to conjure the ‘Ghost in the Machine’ once again” (2012: 401), as if it could be given to a machine and, equally, be taken away and transferred to a different machine. Realizing the robotic installation, *the Articulated Head*, the authors found that, instead, “agency cannot be instilled; it needs to be evoked” (2012: 401). To avoid pre-scripted behaviors, Kroos et al. developed an attention model, which plays a central role in a tightly coupled perception-action control system (2014). Following the sounds in the robot’s surrounds and tracking visitors’ faces, the installation’s resulting attentive behaviors are sometimes reminiscent of Edward Ihnatowicz’s pioneering cybernetic sculpture *the Senster* (1970; see Zivanovic, 2019). In the remainder of this article, I will explore how movement qualities can scaffold a robot’s ability to actively participate in the dynamic meaning-process of an encounter.

## Difference in Relation: Machine Movement Lab

**FIGURE 1:**
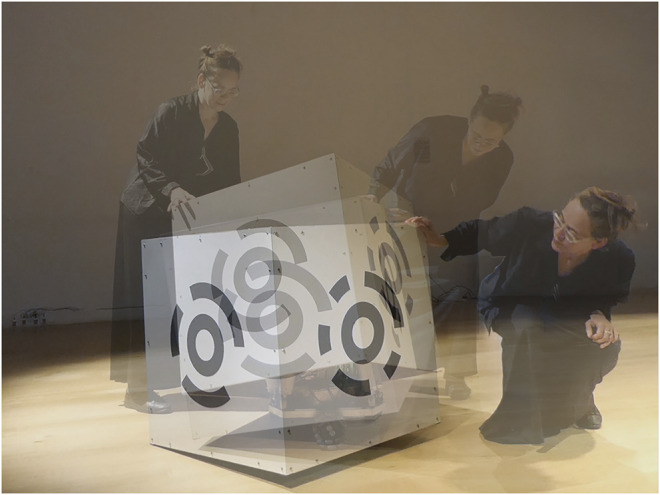
MML *Cube Performer #1*, robotic prototype (composite), Games and Performing Arts Festival, United Kingdom, 2018.

My collaborative *Machine Movement Lab* (MML) project (Figure 1) develops an embodied methodology for designing abstract social artifacts to investigate the aesthetics of meaning-making in human-robot interactions by looking at difference in relation. To illuminate some of the core ideas that motivated our methodological development as part of MML, I would like to return to the question of how a robot becomes a social agent. One could argue that whether or not a robot’s social agency is given (e.g., by the designer) is inconsequent and, instead, what matters is that it is perceived as a social agent by human interactors. After all, I also said earlier that, from a performative view, what we perceive to be agents is an effect of agential enactment between humans and nonhumans. So why does it matter what or who gives rise to a robot’s sociality—do not both approaches produce the same effect and, accordingly, the same possible relationships we can have with robots? I argue that it *literally* matters how we approach the design of social agents, beginning with how we imagine our relationships with them^5^.

By understanding social agency as an attribute, it follows that these social qualities need to be defined in ways that allow them to be represented as part of the design of social agents, whether in the form of physical features or programmable capabilities. This requirement quickly connects us back to Mindell’s argument that it is the designer’s beliefs and intentions that shape a robot’s capacities and, consequently, the relationships it brings about (2015). While this is the case with any designed product, the argument gains more significance within the context of artificial social agents, fabricated to tightly integrate into our societal fabric. Beliefs and intentions do not only manifest in marketable, communication-friendly features but also material boundaries in terms of what they include and exclude and, more importantly, *whose* agencies they affirm, extend, omit, or inhibit.^6^ Other relevant potential dividing lines that our beliefs manifest through practice relate to how we differentiate between human/nonhuman, mind/body, subject/object, as well as information/matter. Hence, one major assumption shaping our interactions and how we imagine them is whether we believe that these dualisms and the hierarchies they impose are the result of an inherent difference between them or whether they are constructs, enacted and reaffirmed as part of a history of epistemological and ontological practices.

In a performative view, “[a]gency is not an attribute but the ongoing reconfigurings of the world” (Barad, 2003: 818) that our discourses and practices are an inextricable part of (as is matter itself) and it is these ongoing intra-actions that differentially enact boundaries, properties, and meanings (Barad, 2003). Designers and engineers find themselves in the midst of a dynamic meshwork of configurings, and it is impossible to avoid boundary-making or material manifestations of our assumptions. Instead, the goal of my relational-performative design approach is for capacities and boundaries to be negotiable in the encounter, that is, to “give space” to the unfolding of a robot’s social capacities and relationships (see Mindell, 2015) in the interactional dynamics instead of pre-shaping them. A relational-performative process, as developed in MML, thus shifts the design focus from designing an “agent” to exploring human-machine couplings (Alač, 2011) and probing into the dynamics through which social agency can emerge in a particular situation. Looking at how an artifact or machine becomes an agent from within the dynamics of the encounter, I argue, challenges rigid subject-object boundaries and renders them more elastic (explored further in the following sections). Aesthetics here does not serve to make the strange look more familiar but is about *rendering differences relational.* The following provides an account of performative meaning-making as it unfolds in our practice, with the esthetic goal to create a rich playground for investigating difference in relation at work by embracing and playfully exploiting the differences between human and machine.

### Machine Movement Lab

Starting in 2015, MML brings together creative, embodied practices with robotics and machine learning, grounded in an enactive, performative framework. The project’s main objective is to open up alternative pathways to social robot design by investigating the relational, generative potential of movement qualities for meaningful encounters with abstract, social artifacts. The question that guided our methodological development was how can a robot with its unique “machinic” differences become relational and participate in social encounters without tightly orchestrated predefined tasks that would prescribe the social scenario? Movement was identified very early on as being key to transforming the social potential of abstract artifacts (see Levillain and Zibetti, 2017). Instead of looking at movement as a medium for “accurately expressing the robot’s purpose, intent, state, mood, personality, attention, responsiveness, intelligence, and capabilities” (Hoffman and Ju 2014: 91), however, MML focuses on the potential of its “distinctive spatial, temporal, and energic qualities” (Sheets-Johnstone, 2012: 49)—qualitative dynamics that cannot only be observed but also kinaesthetically and empathically felt (Sheets-Johnstone, 2012; Despret, 2013; Fuchs and Koch, 2014; Koch, 2014; Gemeinboeck and Saunders, 2018).

The proposition is that the effective, sociocultural dimensions embedded in our movement qualities can serve to bootstrap the robots’s learning to situate it in “the social and cultural scaffolds that human embodied beings are situated within” (Lindblom, 2020: 4). The latter are, according to Lindblom, “the driving force for the emergence of our embodied social understanding” (2020: 4). Importantly, the robot’s learning also needs to be grounded in its own unique, machinelike embodiment, and the human movement qualities require transformation that responds to the differences of this other embodiment. To investigate this potential, we developed an embodied mapping approach, *Performative Body Mapping* (PBM), which harnesses the expertise of dancers to, essentially, “train” a robot to develop sensibilities for human movement qualities (in the form of learned biases and constraints) without simply reperforming human movements. The goal is not to train the robot as if it was a human dancer; rather, we aim for unscripted, embodied meaning-making encounters with an improvising robotic artifact that has learned a few tricks from a human dancer. But “learning tricks” from a human dancer is a challenge for a simple object without legs, arms, a spine, or head. In fact, we did not know how the object would look like at the start of the process, as we wanted to begin with the movement potential itself, rather than a given shape that has to learn to move.

**FIGURE 2:**
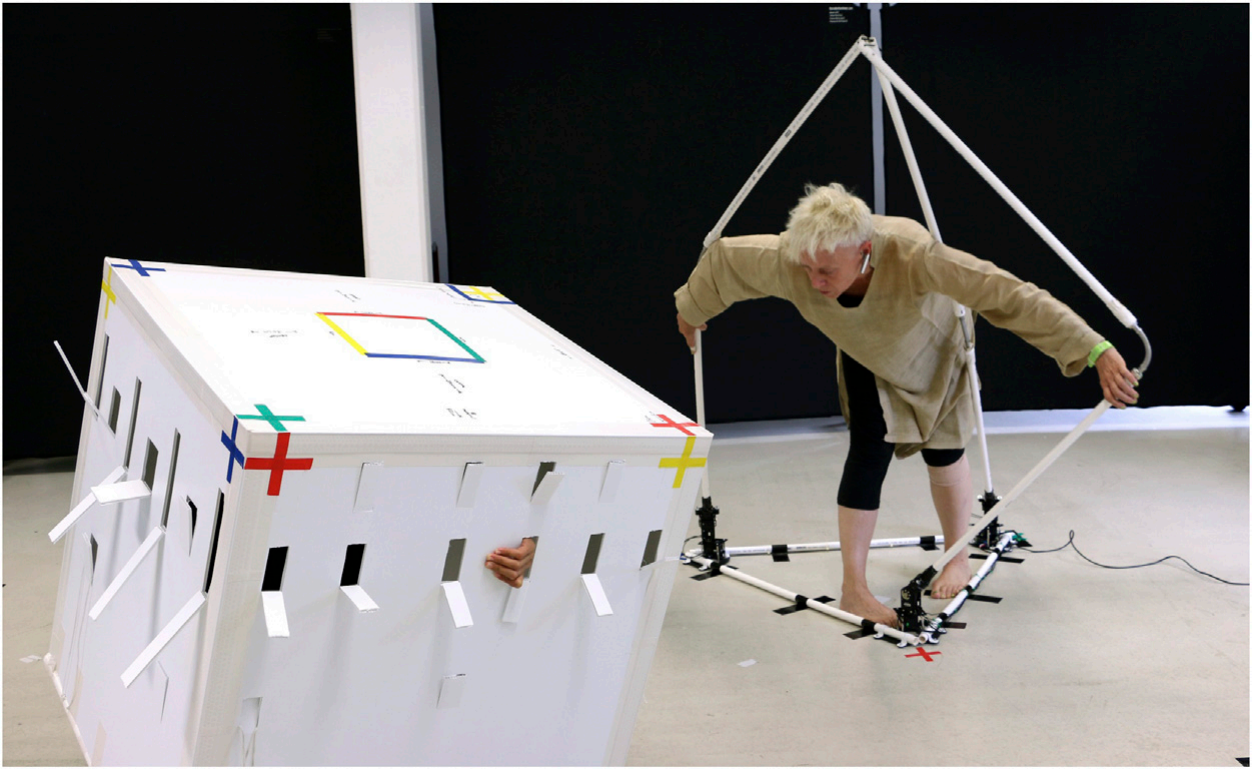
PBM performer-cube and performer-tetrahedron entanglements, with T. de Quincey (right).

The first PBM research stage (2015–2018) began with a series of experiments that involved dancers becoming entangled with a wide range of materials to kinaesthetically feel and extend into other nonhuman forms and their material affordances. We then selected two simple geometric shapes, previously inhabited by performers to study the transformative potential of movement qualities, to take on the role of “costumes” that stand in for the real-size shape of a becoming-robot (Figure 2).^7^ Combining the ideas behind theatrical costumes (Suschke, 2003)^8^ and demonstration learning in HRI (Billard et al., 2008), the PBM costume enables dancers to “step into” and inhabit this other nonhuman embodiment to 1) corporeally experience this strange morphology and learn to kinaesthetically extend into and move *with* it, and 2) bypass the correspondence problem (Dautenhahn et al., 2003).^9^ This is significant because the PBM costume allows 1) delegating much of the difficult morphological mapping to the movement expert and 2) the robot prototype to learn from the motion capture data as if it was trained by another robot performer with the same physical shape. The first stage (PBM) focused on the transformative potential of movement qualities and intra-bodily resonances (see *Bodying-Thinging, Designing with Bodying-Thinging*), while the second, current stage (Relational Body Mapping, or RBM) involves the robot’s unique sensorium to explore how movement qualities transform the relational space between different agents, including artifacts, human performers, and the surrounds (see *Dancing with the Nonhuman*).

PBM aims to tap into our bodies’ tactile-kinaesthetic capabilities to develop and recognize “the synergies of meaningful movement” (see Sheets-Johnstone, 2010) to exploit one of the most interesting characteristics of robots, from an embodied meaning-making perspective that we can bodily resonate, kinaesthetically extend into, and relationally make meaning with their spatial, embodied dynamics and the relations they spawn. Working with choreography, PBM allows us to create a library of qualitative movement dynamics with dancers’ “tactile-kinaesthetic bodies” Sheets-Johnstone, 2010) from within the different material-relational perspective of the robotic embodiment. This bodily inventive process, in Noland’s words, “entails nothing less than the performative construction” of the dancer’s body (2009: 1). We deliberately do not work with narratives or emotional states but, instead, performers often use mental images of nonhuman dynamics, e.g., reimagining their body as a distributed nervous network,^10^ to guide the reconfiguring of their bodies and finding of new movement patterns.

**FIGURE 3:**
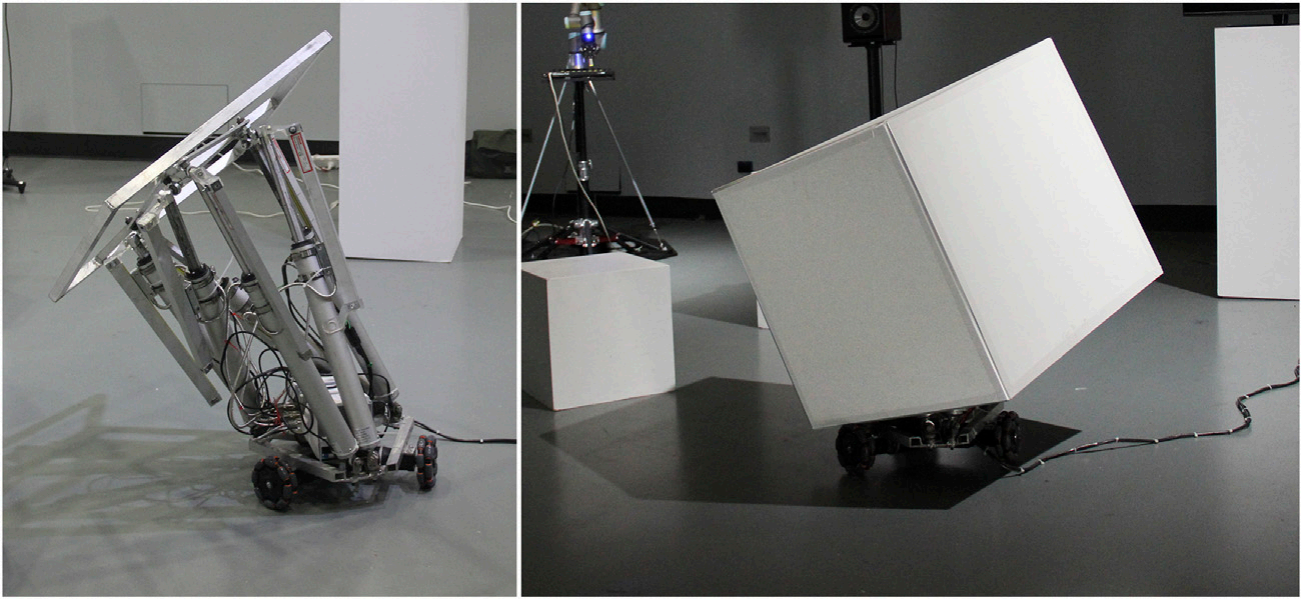
PBM *Cube Performer #1,* robotic prototype (right), and its mechanical frame (left), Sydney 2017.

**FIGURE 4:**
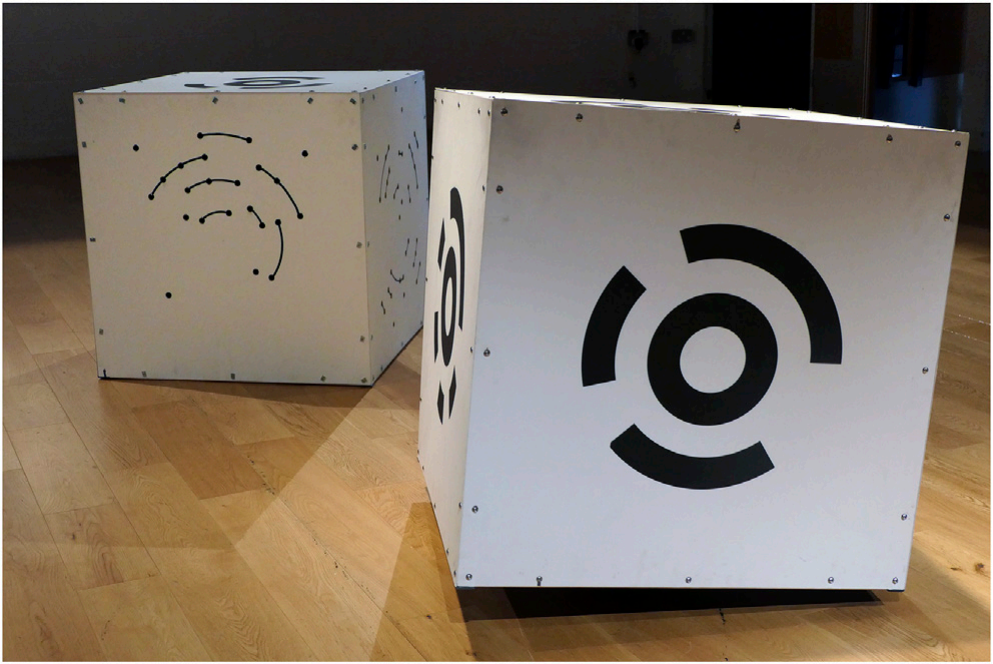
PBM cube costume with *Cube Performer #1,* robotic prototype (right), Games and Performing Arts Festival, United Kingdom, 2018.

So far, we realized one of the costume bodies as two robotic prototypes, *Cube Performer #1* (Figure 3) and *Cube Performer #2*) as part of our iterative design process^11^ (I will look at how we arrived at this particular, familiar shape in more detail in *Designing with Bodying-Thinging*). The Cube Performer has exactly the same dimensions as the cube costume (75 × 75 × 75 cm; see Figure 4) because we consider the scale of the artifact’s shape to be an important part of its material embodiment and the spatial relations it can bring about, and, with regards to PBM, it matters that the two align. We derived the movement requirements for their mechanical design from an analysis of over 10 hours of motion capture recordings to determine the required degrees of velocity and acceleration, as well as ranges of movements—vertically, horizontally, and rotationally (Gemeinboeck and Saunders, 2018). Being essentially plain cube objects, we conceived their mechanical structure to permit changing the outer “skin” of the cube to allow them, like a performer, to integrate into different environments and contexts of encounter (see *Designing with Bodying-Thinging*). It is worth pointing out that the purpose of these prototypes is primarily that of a *materialized, situated research proposition* that allows us to 1) inquire into the potential of relational-performative aesthetics for human-robot encounters and the possible human-machine couplings they entail and 2) develop human-nonhuman performance scenarios to engage publics in important questions about human-robot relationships (see *Dancing with the Nonhuman*).

### Humanlike or Machinelike

Designing with a focus on human-machine couplings, I recognize that the polarization of humanlike vs. machinelike is not helpful and in the following attempt to outline in what ways I understand and use these terms both in our MML process and in this article. In my conversations with choreographers and dancers, I often refer to terms such as humanlike or machinelike to delineate between human body configurations or (habitual) human movement patterns and mechanical configurations or the precise steadiness of robot motion. But, over the course of our PBM movement studies, “machinelike” would also come to denote our “destination,” that is, no longer standing in for typical machinic motion but for movement characteristics that emerged in conjunction with the robot’s spatial-material affordances, activated by a performer-in-costume. With respect to robot design, I use “humanlike” to refer to a designer’s deliberate intent to mimic the human as well as to identify the moments in our process in which we slip into ascribing humanlike qualities to movements performed by the performer-cube entanglement. As our objective is to give space to emergent qualities and unexpected meanings unfolding in the encounter (see also Levillain and Zibetti, 2017), we seek to avoid deliberate inscriptions of specific human meanings or intents as part of the design process. Possible “machinelike” inscriptions that, within the context of our PBM process, are specific to our interpretations of the machine embodiment and its performative potential do not seem to confine the space of possible enactments in the encounter. On the contrary, the blank canvas offered by the plain, regular shape when juxtaposed with a rich variety of movement qualities, seems to open up a wide space for potential “spatial transformations that can be interpreted as actions” (Levillain and Zibetti, 2017: 5) as part of the meaning-making process in the encounter. The results of our participatory studies support this observation, with participants reporting, on average, that they perceived the robot as machinelike yet also evocative, affective, and spontaneous.^12^

While aiming for giving ample space to the enactment of potential meanings in our design process, it is important to note that it is not our intention to avoid anthropomorphic interpretations as part of interactors’ embodied meaning-making process (Levillain and Zibetti, 2017; Airenti, 2015; Hoffman and Ju, 2014; Gemeinboeck and Saunders, 2018). Our aim is for the robot’s performed movement qualities to serve as an empathic-affective scaffold (see Koch, 2014) for interpreting and making meaning while preserving the robot’s unique otherness. My concept of *bodying-thinging* (*Bodying‐Thinging*) directly speaks to the potential effects of this juxtaposition. Hence, rather than aiming to control or channel participants’ interpretations, my relational-performative approach seeks to emphasize the significance of allowing the space for sociality and meaning to be enacted in the encounter, which naturally includes unexpected interpretations and responses (Levillain and Zibetti, 2017).

## Bodying-Thinging

Returning to the question posed at the beginning of this article, this section explores how an *abstract robotic artifact* becomes an evocative participant in social encounters. Levillain and Zibetti (2017) asked a very similar question, looking at behavioral objects and how they trigger human attributions of animacy. While cognitive psychology takes its viewpoint from the human “side” of the encounter, does positioning oneself “in the middle” offer alternative relations? Notwithstanding that our (human) perception, social expectation, and interpretation (Levillain and Zibetti, 2017) play an important role in our meaning-making processes, in Barad’s agential realist account, “meaning making is not a human-based practice, but rather a result of specific material reconfigurings of the world” (2007: 465). A performative perspective on how subjects and object are enacted, in tandem with embodied meaning-making, allows us to not only revisit this divide but to also look at the esthetic, transformational potential of (what we know as) subjects and objects encountering and reconfiguring each other.

The Cube Performer, for instance, brings together the thingness of a “thing” and the dynamics, resonance and affects that “bodies” commonly engender, which seems to suspend the artifact in a position between the two—a thing-body or a body-thing. Looked at closer, this is not a fixed position between the two but rather an ongoing differing—a *bodying-thinging*: a thing becoming more body and the “more body” becoming “more thing” again, and so on. But “bodying” here is not what a body does nor a “thing becoming body.” “Always triggered relationally” (2014: 42), according to Manning and Massumi, it is movement that “bodies-forth” (2014: 39). Rather than a body (or a thing), it is movement in its dynamic differing that is “bodying.” And, neither is “thinging” done by what an object represents. In his seminal text on “the Thing,” Heidegger states, “[i]f we let the thing be present in its thinging from out of the worlding world, then we are thinking of the thing as thing” (1971: 178), untied from an object’s utility. As (human) interactors bodily empathize with the artifact becoming more than what the object represents, they are *bodying‐thinging* in resonance with the *bodying‐thinging* of the robotic artifact. Despret, writing about how we seek to understand animal behavior, describes this transformative bodily reading and communicating as “‘undo[ing] and redo[ing]’ each other” (Despret, 2013: 61). The boundary reconfiguring, that is, *bodying-thinging* thus allows (human) interactors to corporeally resonate and respond to a dynamically moving artifact, whose embodiment and behavior are very different from their own. Intelligibility here is not confined to matters of intellection but “rather more generally may entail differential responsiveness to what matters” (Barad, 2007: 470). In Despret’s concept of “embodied empathy” (2013), this ongoing attunement is reciprocal but not symmetrical and always only partial. Hence, *bodying-thinging* is not about turning objects into subjects or the other way around but about bodily making meanings across the subject-object divide and rendering elastic fixed boundaries in the process.

*Bodying-thinging*, I propose, is a form of *entangling*—how subjects and objects entangle and are transformed in the process of human-robot encounters. Entanglements, in Barad’s words, “are not unities. They do not erase differences; on the contrary, entanglings entail differentiatings, differentiatings entail entanglings” (2014: 176). Writing about machine performance, Dimitrova identifies a “constitutive connectivity [that] allows bodies to become dissipative structures,” an empathic “being toward” that allows us to “peer into regions that were previously unintelligible” (2017: 175). Yet, this potential for connectivity is not restricted to organic bodies (see Dimitrova, 2017) but is also how bodies and things entangle and subject-object boundaries become porous, opening up mutual intelligibilities.^13^ In my relational-performative view, machines are no longer positioned outside the social and do not need to be given sociality with, for example, a humanlike veneer. As we will see in the following section, *designing with bodying-thinging* is the playground of a relational-performative aesthetics, mobilizing and embodying our attention to causal enactments and their “ongoing differentiating intelligibility and materialization” (Barad, 2003: 824) that we participate in, designers and participants alike. Dance and choreography are natural allies for bodily inquiring into human-nonhuman encounters and how they *reconfigure relations*, the kinds of which that induce forms of *bodying-thinging.* Dancers are extremely attuned to their body’s ongoing reconfigurations in relation to space, other bodies and things, and more so, can be highly skilled to tune into and reconfigure other bodies and things. This relationality, affectively *being toward* through movement, is described by Leach and deLahunta, 2017 as “an extension of feeling, knowing, and sensing into the world *with*, and of, other bodies” (2017: 464). In the following, I will take a closer look at PBM and how the dancer bodily and kinaesthetically tunes into the otherness of a robot’s embodiment through the PBM costume.

**FIGURE 5:**
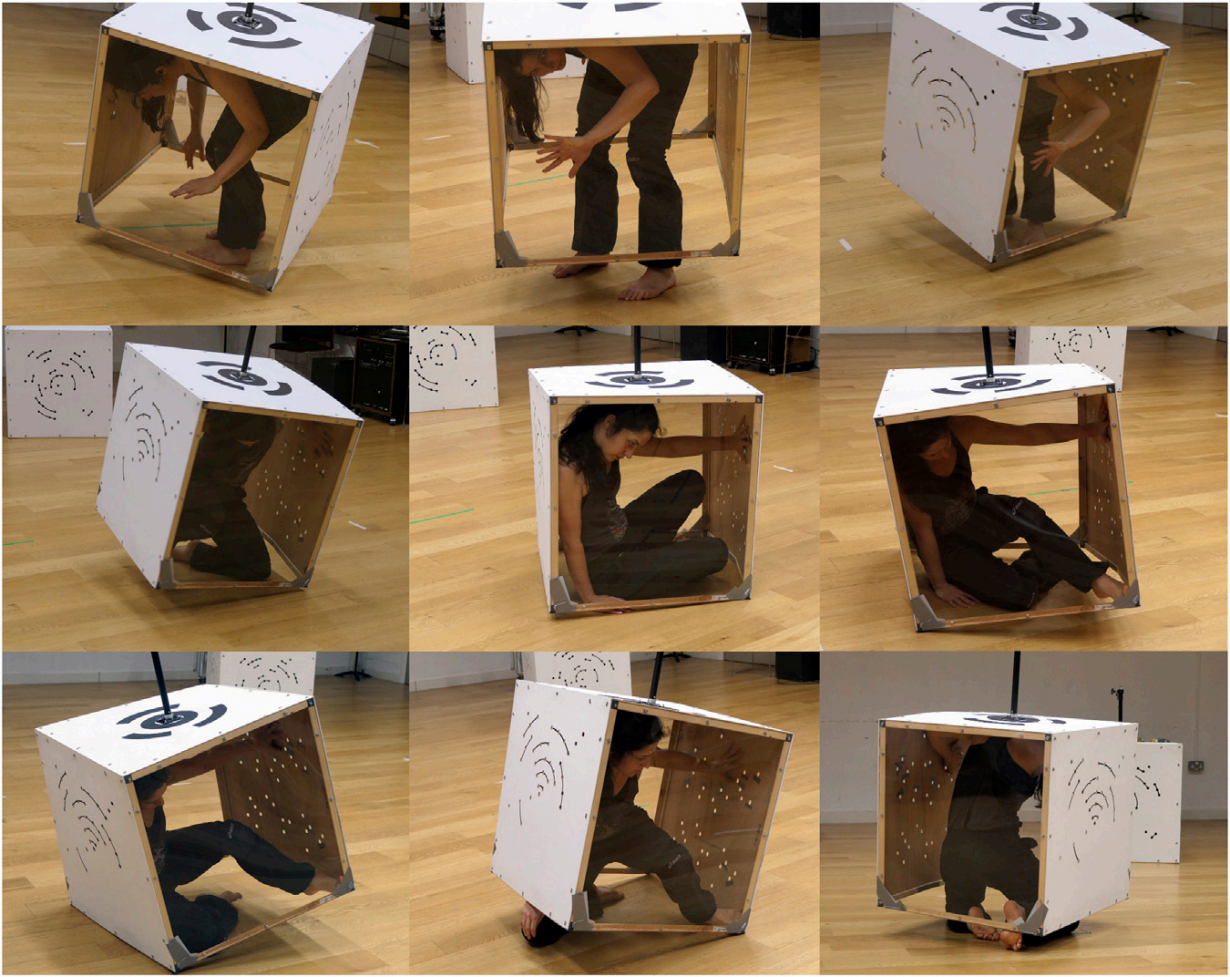
PBM performer-cube entanglement in sequence, with A. Rochette.

The esthetic, performative differing of *bodying-thinging* not only frames the process of how subject-object boundaries are continually enacted and transformed in an ongoing “undoing and redoing” each other, but in PBM also materially manifests in the performative mappings. PBM harnesses dancers’ ability “to become virtuosos of coping,” which means to become, in Noland’s words, “experts at adapting their own sensorimotor instrument to the situation at hand” (2009: 1). According to Noland, technology in choreographic contexts serves to establish environments and situated demands that challenge dancers “to discover the ways human bodies produce themselves (how they refine their capacities and thus assume new shapes)” in relation to these demands (2009: 1).^14^ This relational bodily reconfiguration perfectly encapsulates the *bodying-thinging* that goes on in the PBM performer-cube entanglement. A dancer, who has inhabited the cube costume^15^ for over 30 h, described her coping response to the challenge posed by the costume as her “body extending into the cube.” To “give weight to the costume,” for example, she needs to assume different shapes that afford her “to transfer tension by pressing against [the costume’s] surfaces”^16^ (Figure 5). Coping here is about skillfully intra-acting with the environment and its relational affordances, rather than controlling what might else be seen as a passive “container.” By “extending into the cube,” the dancer does not impose her body onto the artifact but rather “becomes body-thing” with the cube. Importantly, as she becomes a virtuoso of this intra-active coping, her proprioception also transforms to afford her to kinaesthetically sense her body-cube entanglement and its movement and position in space.^17^ It is this bodily-kinaesthetic probing and puzzle-solving (see Noland, 2009) and how it performatively reenacts bodies and things as relations of *bodying‐thinging* that is at the heart of my relational-performative aesthetics.

*Bodying‐Thinging* in PBM is not a simple transaction like the human dancer “bodying” the machine or the machine “thinging” the human dancer. Bodying or “bodying-forth” denotes to the relational meanings produced by the machine’s differing through movement dynamics—a being toward that we usually connect with the lifelike or animated. Importantly, movement here is not only about couplings between bodies and the environment but also brings with it cultural and historical couplings (see Gamble et al., 2019). By “thinging,” the machine brings forth its “thingness,” an otherness unique to the machine and the relations it constitutes and is constituted by, commonly in conflict with notions of the animated. Yet, according to Heidegger, the “thinging of the thing” also brings forth a “nearing,” a nearness being “at work in bringing near” (1971: 175). While entangled, based on our observations of how performers, choreographers, and participants make sense of the encounter, *bodying-thinging* has the effect of rendering the artifact in motion *at once stranger and more familiar.* For example, in one of our PBM experiments, guided by the image of breath and how it changes according to different bodily states, the performer-inside-costume balanced with the cube on one corner, while raising the diagonal corner using varying qualities of velocity, rhythm, projection, and weight. Naturally, neither the costume cube nor the robotic cube is perceived as “breathing” as a result. However, the dynamic motion patterns that they perform arising from the dynamic qualities brought forth by this image render both the costume and the robot’s machinelike performance of it stranger and more familiar at the same time. The effect of this affective juxtaposition has been expressed by one of our study participants^18^ as “I like its non-humanness … there is a companionability to it. Wow.” Another talked about approaching it “as a subject but then it flips around and does something else.” Participants also described their intra-bodily resonances in ways that parallel their relationship with nonhuman animals, saying, “I responded to it like another species and increasingly so” or “it comes across as playful … like a wild animal.”

Writing about our encounters with companion species, Haraway poignantly observes that embodied communication “is more like a dance than a word: the flow of entangled, meaningful bodies in time—whether jerky and nervous or flaming and flowing, whether both partners move in harmony or are painfully out of synch or something else altogether—is communication about relationship, the relationship itself, and the means of reshaping relationship and so its enacters” (Haraway, 2008: 26). We *body-thing with* the artifact.^19^ The resonances felt as one is entangled in this dance are, I argue, not only one-sided projections or attributions that bestow either animatedness or thingness onto an artifact,^20^ rather meaning arises with respect to how the artifact actively extends toward us—how it *body-things*—and how this “intra-bodily resonates” (Froese and Fuchs, 2012: 212) with us and we extend toward it in response. Affective and agential effects here arise from the entangling of *bodying-thinging*, rather than individual control or one-sided projection.

## Designing With Bodying-Thinging

As we shift from a representational view, anchored to distinct entities, built-in agencies, and fixed boundaries to a relational-performative approach, design as a practice becomes part of reconfiguring the world (even if only a very small part of it). Entanglements with the nonhuman not only involve (preformed) objects and machines but matter itself and the different sociomaterial relations it is embedded in. A relational-performative design approach is thus more akin to humbly participating in the “ongoing open process of mattering through which ‘mattering’ itself acquires meaning and form in the realization of different agential possibilities” (Barad, 2003: 817). Meaning-making here happens as part of a process of embodied, situated material engagement (Malafouris, 2013), where materiality is not a matter of representation but rather of capacity and relationality—what it *does* and how it can *participate in* the wider meaning-making context. This inseparable entwinement of embodied, material engagement, agential enactment, and meaning-making is core to our MML design process. Its embodied attentiveness to the relational in meaning-making also aligns with Dourish’s (2001) embodied interaction approach to HCI design that offers a method of attending to the situated, social aspects of meaningful embodied encounters. Embodied, material engagement and relational meaning-making thus not only play key roles when a robot is “ready for relationships” (Turkle, 2005: 288) but are equally core to the design process. This is where the material relation-making begins.

Our human-robot encounter begins when we imagine the robot, experiment with its design, prototype it, and share, evaluate, and make meaning of its *bodying-thinging* and *body-thing* with it. This is how we can *design with* its relational potential and the dynamics it arises from, along the process, rather than encountering the robot for the first time at the end of the design process. In such a performative process, the design brief for a robot is never complete, because the intra-active process of both design and encounter (i.e., human-robot interaction) constitutes and continuously reconfigures capacities for relation-making. In order to give space to the enactment of relations and the making of meaning in the encounter, I propose that the two distinct processes of design and encounter need to be understood as one continuous process—*designing with* the encounter.^21^

### Giving Space to Bodying-Thinging

The difference of *designing with* the encounter (rather than *for* the encounter) is how we attend to 1) the agential networks that nonhuman materialities are embedded in, as well as 2) the agencies and meanings that our process inevitably makes manifest in the design, whether deliberate or unwittingly, and 3) how these (1 and 2) intra-act with the meaning-making, later, in the encounter with participants. Every design process necessarily involves decisions that include certain possibilities and exclude others and that eventually manifest in specific material forms and behaviors (see also *Difference in Relation*). Let me briefly introduce some stages of our process and how it was propelled by the transformational potential of relational movement qualities and material affordances.

**FIGURE 6:**
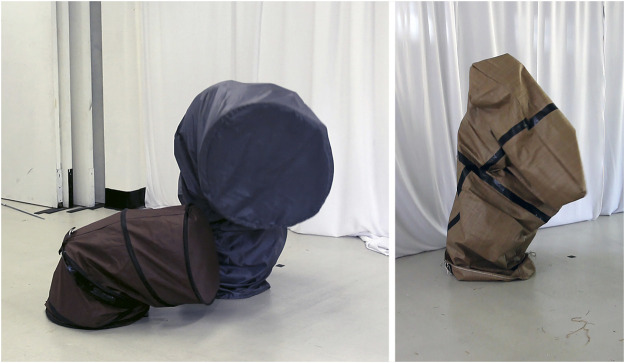
PBM material studies with fabric costumes, inhabited by performers.

**FIGURE 7:**
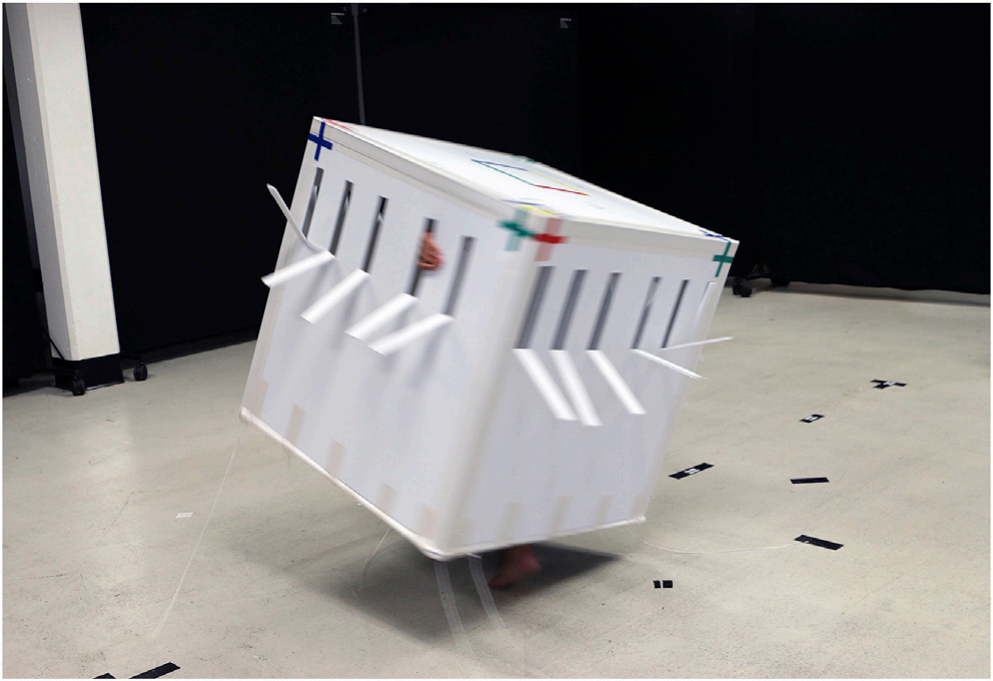
PBM performer-cube entanglement in motion.

Our PBM design process began with *unfixing* relations by materially investigating them, rather than focusing on tasks and their potentially already fixed relations. Doing so, our process started with a series of human-nonhuman encounters, with dancers bodily probing into the performative potential of a wide range of material relations. This early stage, which favored movement and relationality over visual characteristics, focused our esthetic approach on investigating the potential of “disjunction of form and movement” (Bianchini and Quinz, 2016). Continuing, we began to bodily probe into the transformational potential of simple geometric shapes in motion, e.g., cylindrical, cuboid, cubic, and tetrahedral forms, made of qualitatively different materials, e.g., stiff, elastic, and springy (Figure 6). While materials with a built-in kinetic capacity, once activated by a dancer, made for an interesting and playful process of generating continuously changing objects, their transformational potential was dominated by physical transformation (e.g., folds, twists, and stretches), rather than relational movement in space. Fast-forward through a few more weeks of embodied experimentation and this is how we arrived at the cube^22^—first as costume and later as robotic artifact. A cube presents a highly abstract yet familiar form, which, on its own, is not usually considered to be expressive or having a social presence. Looking at body-space relationships, a cube’s regular, symmetrical, and omnidirectional geometry counterposes organic structures (e.g., human) with limbs, two-sided symmetries and the hierarchy of front and back.^23^ Most familiar when sitting flat, rooted in place, a dynamically or delicately moving cubic object, suddenly tilting up, gently swaying. or rambunctiously thumping onto the ground, quickly loses its rootedness and, with it, its stability (Figure 7). Hence, a mechanical cube, learning from a human dancer, performs the disjuncture of plane, regular appearance and intricate, dynamic movement. The thing becomes a body-thing, transformed through the relational dynamics of movement.

The latter could suggest that the dancer inscribes the machine with her human intent as she bodily extends into the costume, whose motion-capture recordings seed the machine learning. Indeed, it is the *bodying-thinging* of the performer-cube entanglement through which the framing of possible human-machine encounters and the potential for relational meaning-making is beginning to take shape, quite literally. Rather than aiming to inscribe the robot with human intent in the form of representational gestures (McNeill, 1992) or narratives, we work with choreographers and dancers to socioculturally situate (see Lindblom and Ziemke, 2003; Lindblom, 2020) the abstract artifact by bootstrapping its learning process based on qualitative movement dynamics. Importantly, these movement dynamics arise from choreographic abstractions (see below) or improvisations in which the dancer entangles with the “other body” of the machine to perform the identity of this other, machinelike “body”; through this entanglement, the performer is tasked with exploring the enabling constraints of the costume rather than anthropomorphizing or imprinting themselves onto the cube. Our motion data then capture the kinetic dynamics of the performer-cube entanglement and comprises granular, discrete movement patterns, derived from short choreographic abstractions, that is, movements that can only be observed “as movement *per se,* for the sake of motion itself” (Aviv, 2017: 4). In the machine learning process, this catalog of dynamic movement qualities serves as aesthetically and socioculturally coded biases and constraints. The robotic artifact learns to compose new movements based on these learned biases and constraints, but, importantly, its learning has also been grounded in its own unique material embodiment. I thus put forward that the Cube Performer’s relational potential is shaped by the dynamic movement qualities that the machine performs, situated in the sociocultural, human context of the research team. In linking back to Haraway’s statement that communication is more akin to dance than a word (2008), seeding the material-digital process of the robot learning to move with abstract relational patterns that we know how to corporeally “read” or intra-bodily resonate with affords the machine participant to *dance with* its human interactors. Yet, the familiar patterns not only get displaced as they are digitally mediated but also transform in the machine-grounded learning process and the robot’s mechanical performance. Movement data here also are *body-things*.

Simon Penny’s artwork *Petit Mal* (1989–2005) opens up another esthetic approach to exploring how a machine actively participates in the encounter, showing how its relational potential arises both from its unique machine embodiment and the dynamics of the particular situation it is embedded in. A pioneering example of a machine performer, *Petit Mal*, appears neither humanlike nor animal-like and behaves and relates to its world in ways that are unique to its machine embodiment. Resembling a strange, responsive dicycle, the work’s unique behavior results from an eccentric mechanism based around a double pendulum, which brings an unpredictable charming quality to its movements, swaying through the gallery space to “engage visitors in large-scale bodily interaction—a dance,” (2016: 57). In his writings, Penny has long been critical of the dualist computationalist separation of software/hardware and information/matter, in favor of a performative view (2017 and 2011). *Petit Mal* embodies this view with hard- and software developed contingent on one another. What is particularly interesting with regards to the machine’s unique embodiment and ability to engender affect and relationality is the complexity of its movements resulting from, by comparison, a simple mechanism. Based on the artist’s embodied, processual and antirepresentational approach and observations of audiences’ bodily responses^24^, to me, *Petit Mal* is *bodying-thinging*. I am not sure, however, that the artist would agree with me. In tandem with descriptions of *Petit Mal*’s relationality being enacted as part of interactional dynamics, he frequently positions the work as “an autonomous machine” (Penny, 2011: 85; see also 2000, 2016, and 2017), which suggests an understanding of autonomous agency as a condition for participation rather than the effect of its relational network being cut off (Suchman, 2007a). In contrast to my approach here, Penny’s performative ontology of an “aesthetics of behavior” counters notions of entangling and agential enactment in favor of autonomous machines that “make decisions and take actions” (2016: 401).

Narratives of machine autonomy position the artist/designer outside of the ongoing reconfigurings of the world (see Barad, 2003; Stacey and Suchman, 2012) and serve to detach the machine from the designer, its users/participants, and the wider network that the machine and design process are embedded in. In contrast, once we find ourselves inside and part of the ongoing reconfiguring, we are no longer distant or external and can only design *with* the relational dynamics and the contexts they arise from. Suchman talks about the singularity of the interface exploding “into a multiplicity of more and less closely aligned, dynamically configured moments of encounter within sociomaterial configurations, objectified as persons and machines” (Suchman, 2007a: 268). This is the “stuff” that we design *with*.

### Opening Up Spaces for Emergence Through Staging

Staging is an important practice for HRI, allowing a robot to be situated in various sociomaterial and cultural settings and frequently used to promote a robot’s autonomous agency (see Suchman, 2007). While often overlooked as part of the design process, from a relational-performative perspective, staging is in itself a powerful esthetic intra-action that actively sets and shifts boundaries by “systematically foreground[ing] certain sites, bodies, and agencies while placing others offstage” (Suchman, 2007a: 283). If we think of encounters as intra-actional performances (see Barad, 2003; Barad, 2007), then staging is the making of their performance context, giving space to or inhibiting possibilities for entangling (*bodying-thinging*).

**FIGURE 8:**
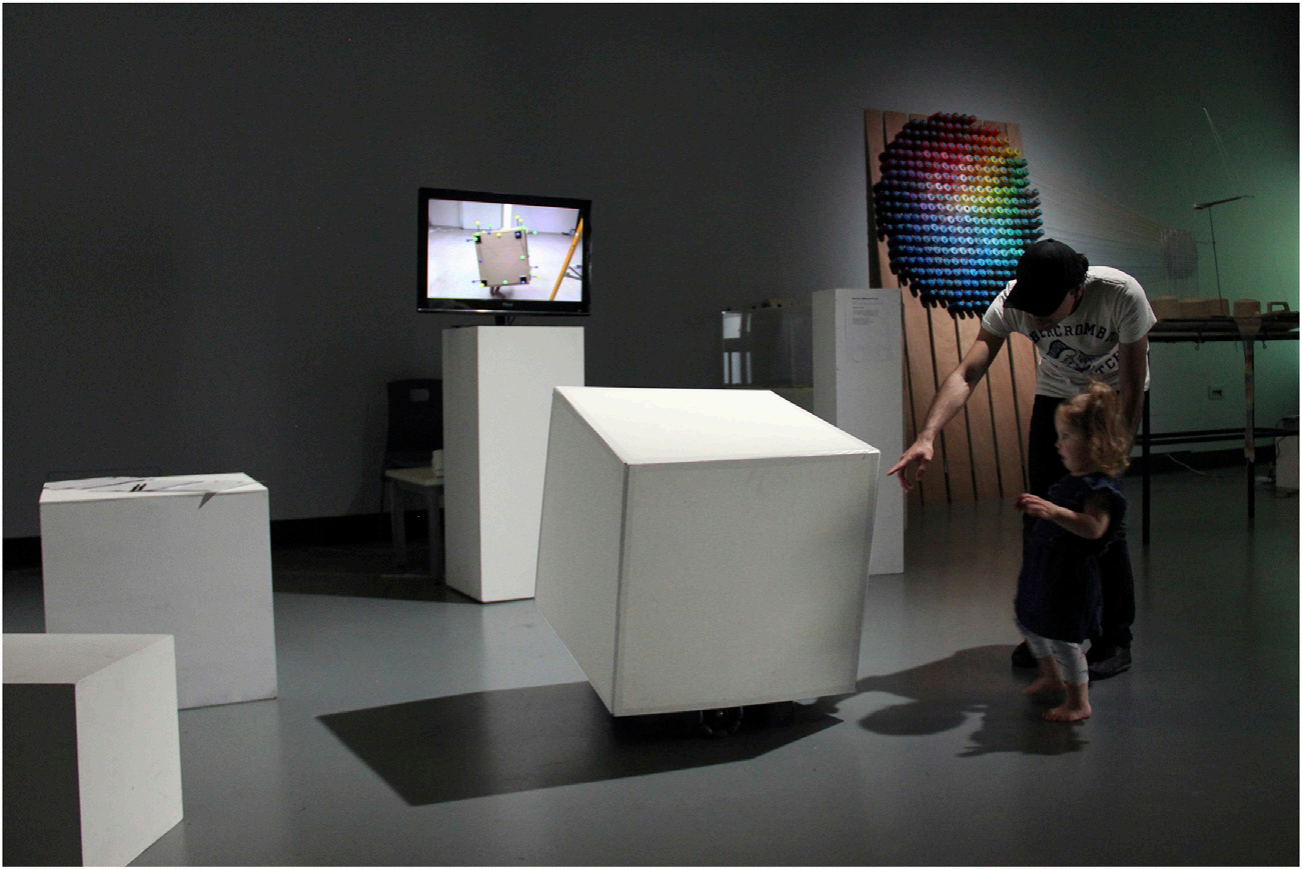
MML *Cube Performer #1*, robotic prototype, at RePair, The Big Anxiety Festival, Sydney, 2017.

**FIGURE 9:**
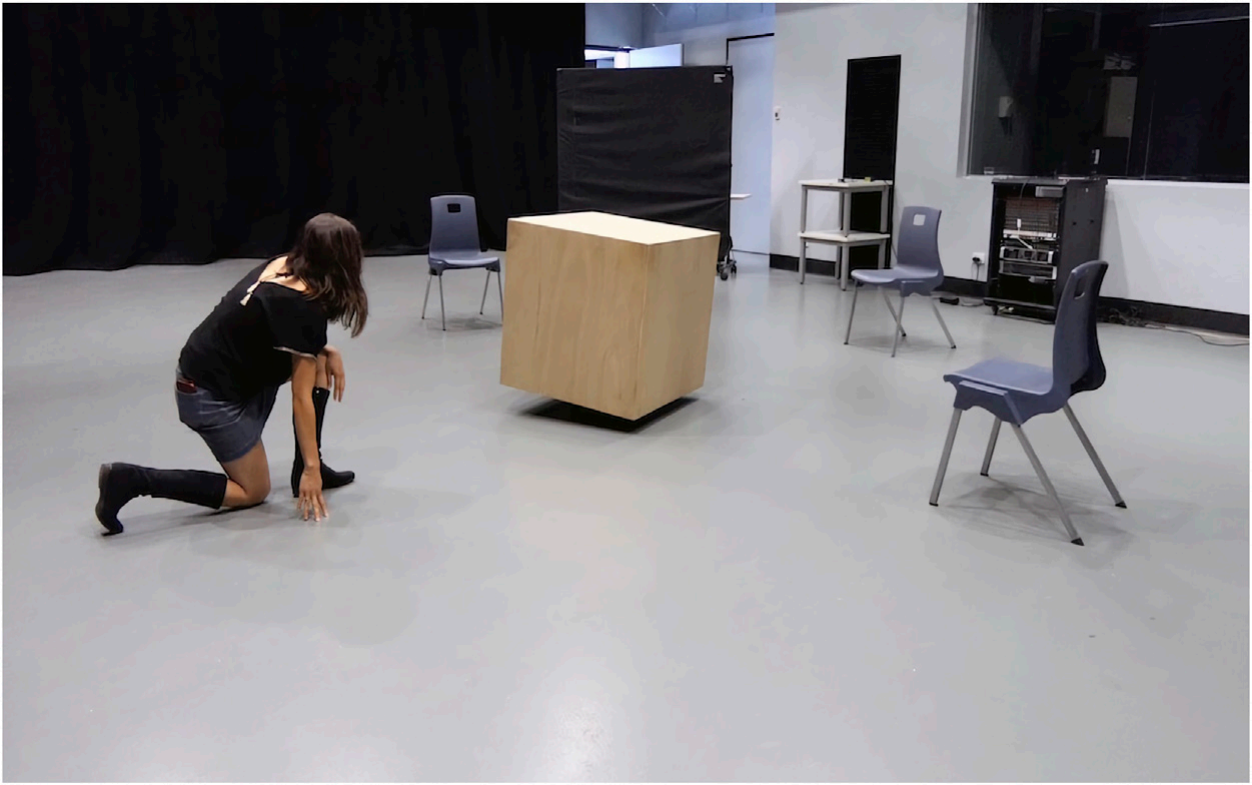
MML Study, participant with *Cube Performer #2*, robotic prototype, Sydney, 2019.

While it can be tempting to exploit our age-old fascination with self-moving machines by staging them as visual spectacles, I have so far approached the staging of the Cube Performer through the aesthetics of the anti-spectacle. This, in my practice, involves nestling the robotic performer(s) into an environment in ways that foreground the unfolding dynamic enactments and how they transform the situation.^25^ As MML has so far focused on methodological development rather than performance-making (see *Dancing with the Nonhuman*), our staging considerations mostly involved how the robot visually integrates in existing (gallery) contexts in ways that heighten its relational potential brought forth by its movement qualities. Importantly, this also meant that we present the Cube Performer as a prototype or “research work in progress” rather than a complete artwork, motivated to show the prototype at different stages of the PBM process to gain insights into whether and how audiences/participants “intra-bodily resonate” and engage with the robot in an unscripted encounter. So far, we have staged very simple first-encounter scenarios with the Cube Performer as part of two public exhibitions,^26^ e.g., integrating *Cube Performer #1* into the gallery context by staging the prototype as a gallery plinth among a group of other (immobile) plinths (Figure 8). In the open-lab study,^27^ without an exhibition context, the robot took on a utilitarian identity to blend into the studio/lab context, appearing like a simple wooden box (Figure 9). These “humble” stagings suited our iterative prototyping stages and the contexts of encounter, particularly since, to us, staging is about preparing the ground (including the looks of the artifact) for giving space to the possibilities generated by the movement dynamics of the robotic artifact. Integrating the artifact in the environmental context worked so well that, at the opening, two audience members jumped when the apparent plinth, which they placed their glasses on, began to twist toward them (more discussion of audiences’ responses can be found in *Discussion*).

**FIGURE 10:**
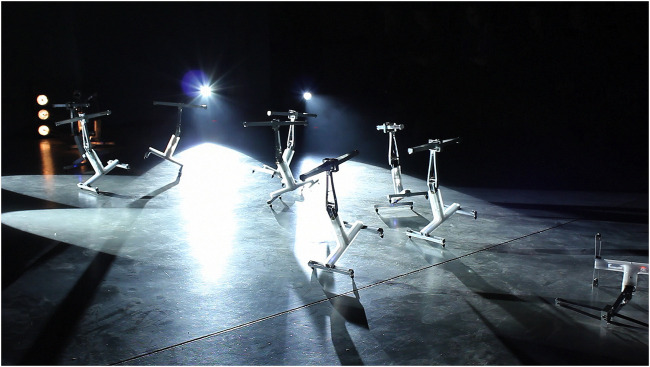
*The Tiller Girls* by Louis-Philippe Demers, V2 Rotterdam, NL, 2010.

Louis-Philippe Demers’ *The Tiller Girls* (2010) is an example of a meticulously choreographed stage work that gives ample space to emergent relations and even welcomes unplanned collisions and tumbles. The work brings into conjunction the cultural legacy of the 1930s (human) dance ensemble, named “The Tiller Girls,” with a troupe of 32 small machine performers. The robots, deployed as machine performers in *The Tiller Girls,* were originally developed by scientists Fumiya Iida, Raja David, and Max Lungarella at the Artificial Intelligence Lab, Zurich, to “study locomotion and gaits derived from simplified morphologies” (Demers, 2016: 281). Demers’ dramaturgy utilizes the movements and their “fairly rich” qualities produced by these unusual morphologies to contrast “The Tiller Girls” human yet machinelike performance. Opposing the highly synchronized lines of the human ensemble, Demers’ performance unfolds through a dramatic staging of simple machines rhythmically hopping and occasionally falling as part of a “structured chaotic ‘improvisation’” (Demers, 2016: 288) (Figure 10). Similar to the aesthetics of the aforementioned “disjunction of form and movement,” this choreography puts to work the performers’ abstract, simple shape to produce a surprising range of unique movement characteristics (Demers, 2016) that give the troupe a dynamic, unpredictable, and whimsical quality. Like Penny, Demers exploits emergent movements of a simple mechanical structure, here an inverted pendulum, to aesthetically explore notions of “intra-bodily resonance” (Froese and Fuchs, 2012: 212). Performance techniques here are employed to investigate how objects transcend their objectness (see Jochum and Goldberg, 2016) by aesthetically exploiting their physical capacities while opening up notions of *bodying-thinging* to historical enactments that shape how we understand bodies. With it, *The Tiller Girls* dynamically enacts a dramaturgy that shows how staging can open up spaces for emergence to shift and unmake boundaries.

Looking at agency and how it constitutes the machine as performer, all three approaches, Demers’s *The Tiller Girls*, Penny’s *Petit Mal*, and our *Cube Performers #1 and #2*, favor movement over morphology.^28^ Yet, we have arrived at three quite different perspectives on how a machine artifact becomes more than an artifact based on its dynamic movement qualities. In Demers’s view, *agency is attributed* to the machine performer by the audiences’ perception (2016). Interestingly, both the artist and the machine performer have to do work to align the performer’s multiple bodies with its behavior: the machine performer has to align its behaviors with its body and the artist then has to align its behavior with its “given social embodiment” (Demers, 2016: 303). This work, I suggest, serves to modulate the audiences’ perception through the quality of the alignment, e.g., diminishing the performer’s presence “[w]hen the body feels animated, mechanical” (Demers, 2016: 303). In Penny’s view, *agency is given* to the artifact through the design/coding of its “sophisticated behavior” that then allows the agent to “take actions” (2016: 401), albeit in *Petit Mal*’s case it is both the artist’s coded behavior and the instable mechanism, which generates the movement qualities, that bestow it with agency (Penny, 2000). And, in my own view, agency is neither the artifact’s nor the audiences’ to give but, instead, *agency is enacted* in the interactional encounter, which the artifact participates in through its dynamic movement qualities. All three views put forward that it is an artifact’s surprising range of unique movement dynamics that amplifies its relational potential in the encounter (see also Levillain and Zibetti, 2017). In both, Demers’s and Penny’s works, this surprising range and unique quality arise from the machines’ dynamic morphological computation, while our Cube Performer enacts these relational dynamics based on PBM’s intermeshing of human and machine’s very different ways of being.

## Dancing With the Nonhuman

In this section, I briefly introduce my current project, Dancing with the Nonhuman, which sits under the umbrella of the MML project but has its own distinct scope and objectives.^29^ Performance practices have a long history of developing new kinds of agential relations, where the making of the work relies as much on nonhuman “things” as on humans, or where agency emerges across human and nonhuman domains based on an intimate collaboration between the two (Gemeinboeck and Saunders, 2016a; Eckersall et al., 2017).^30^ Dancing with the Nonhuman investigates the potential of performance-making as a research practice to embed our Cube Performers and their machine learning in the sociocultural and sociomaterial milieu of a dance studio. One major goal is to create a public performance work that involves nonhuman (machine) performers and human performers and is open to audience participation. Our process is thus concerned with how the Cube Performer becomes a creative machine performer (see Maher et al., 2008) to facilitate co-improvization with dancers and audiences. The approach situates our robot design in the development of a performance-making practice, rather than bringing performance techniques to the development of a robot design practice. The former permits us to explore meaning-making and specific configurations of *bodying-thinging* from the perspective of the encounter as performance event, fusing the esthetic with “the social, political, and ethical” (Fischer-Lichte, 2008: 172).

To situate the machine performer within the continuously evolving performance context, our approach builds on PBM’s embodied mapping interface but opens up to the importance of perception in meaning-making (Noë, 2009; Johnson, 2018) and perceptual learning (Gibson, 1963). To render the Cube Performer a creative machine performer, we are developing an expanded mapping interface that allows us to study the intertwinement of movement, perception, and situated meaning-making.

Like embodiment, a robot’s perception is radically different from human perception, independent of how humanlike or machinelike it might appear. Hence, while humans and robots may physically share a social space, from a biosemiotic viewpoint, they are each embodied in their own unique *umwelt* (Uexküll, 1957; Ziemke and Sharkey, 2001). Hence, meaning-making between humans and robots is an intra-bodily enactment across differentiated ecological niches. To afford dancers an embodied insight into the Cube Performer’s unique machine *umwelt*, PBM’s embodied interface is extended to allow for mapping between human and nonhuman perceptual worlds.

Relational Body Mapping (RBM) expands the PBM costume with an identical set of sensors as those used by the robot^31^ to enable the dancer inhabiting the costume to experience the robot’s sensorium, made “tangible” to the dancer in the form of a dynamic soundscape. The RBM costume thus becomes a performative sensorial mapping instrument for enactive investigations into how movement shapes perception (Noë, 2004; Noë, 2009). The purpose is to study the performer-in-costume creatively working with the asymmetries between the two perceptual worlds and how this affects their relations with the environment and its dynamic affordances and resulting movement qualities. This, then, will allow us to bootstrap the learning of the Cube Performer with the motion patterns of the performer-cube entanglement capturing its qualitative movement dynamics in relation to interactional and environmental affordances (see Gibson, 1979; Rietveld and Kiverstein, 2014) based on negotiated *umwelts* (by the dancer). We are interested in the Cube Performer learning a generalisable model of this situated and perceptually guided (see Lindblom, 2020) motion data, based on the dancer having access to the robot sensorium; I cannot yet speak to whether and how this expands the robot’s improvisational capabilities as, at the time of writing, we are still in the process of developing RBM. The project also includes a research axis focusing on Laban/Bartenieff Movement Analysis (Laban, 1972; Bartenieff and Lewis, 1980) to produce descriptors used in a custom movement notation system and labels for the motion data used by the machine learner (see Karg et al., 2013).

**FIGURE 11:**
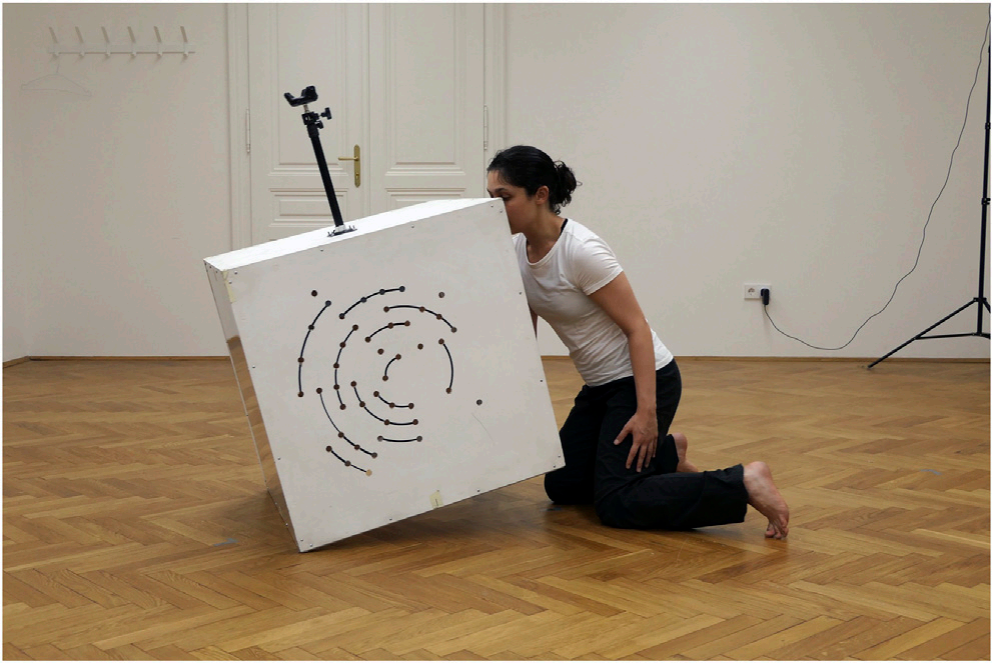
*Dancing with the Nonhuman*, extended choreography experiment with A. Rochette (right).

The first stage of our performance-making process focused on developing a series of semi-improvisational choreographic scores^32^ exploring the creative potential of relational exchanges between human and machine performers. These scores are enacted through embodied exchanges between a performer-costume entanglement intra-acting with another human performer (not in costume) (see Figure 11) and/or other artifacts and machine performers. Our RBM experiments thus expand our previous PBM studies by widening the relational scope to probe into the transformative potential of movement qualities with regards to the relational space between different agents, including artifacts, human performers, and their spatial relationships to a specific context. The relational space in-between agents here is understood as both an emergent result of the interactional exchange and a reconfigurable medium in itself that can be sculpted and rendered elastic through movement and its dynamic qualities of nearness, timing, and amplitude, etc. This tactile-kinaesthetic, spatial puzzle-solving and reconfiguring of bodies and things aesthetically put to work the asymmetries that arise from the different embodiments and perceptual worlds of humans and machines. Finally, our goal for the public performance work is not only to perform and evaluate our performance-making practice but to also become a research tool for involving the publics in reimagining human-machine boundaries and promoting their elasticity.

## Discussion

This article has laid out a framework for *relational-performative aesthetics in human-robot interaction,* comprising a theoretical lens and design approach for critical practice-based inquiries into embodied meaning-making in human-robot interaction. The following takes a closer look at how movement qualities can contribute to human-robot interaction design and then identifies four areas of design challenges before ending with a summary.

### The Relational, Situating Potential of Movement Qualities

In a relational-performative view, meaning-making is a fundamentally esthetic and embodied process, situated and unfolding in the interactional dynamics. Movement with its generative, dynamic qualities here offers more than cueing users “into what actions and interactions are possible” (Hoffman and Ju, 2014: 95). Robots with a heightened sensitivity toward motion dynamics and the mechanical abilities and improvisational skills to use them as building blocks for affective, social coordination could *generate* new meanings *with* their interactors, opening up aesthetically rich, social experiences without relying on predefined personalities, narratives, or tasks. The relational-performative effects of such skills are likely to be particularly useful in dynamic social environments where humanoid robots are too costly or potentially too risky from an ethical viewpoint,^33^ or, simply, where more diverse robot participants are desired.

What bodily immersion, core to PBM and RBM, affords us is a viewpoint from within a specific machine embodiment and tactile-kinaesthetic access to its specific relational-environmental affordances (see Rietveld and Kiverstein, 2014). Techniques from animation (see Hoffman and Ju, 2014) and puppetry (see Jochum et al., 2017) or Wizard of Oz techniques, where interactions with design prototypes are mediated by a human operator (see Sirkin et al., 2016), also permit designers to project themselves into the artifact and offer powerful tools for creating expressive movements. The shapes and objects that can be imbued with life through these techniques can be surprisingly simple and abstract, as demonstrated in the classic animation of Chuck Jones’s *The Dot and the Line* (1965). Animation often requires movement to be defined through static poses (keyframes) that are then digitally interpolated; we can find a similar approach in MIT’s dialogue-free *Interactive Robot Theater*, which animated robot “actors” by transitioning between a set list of poses (Breazeal et al., 2003).^34^ Yet defining movement through a series of static positions misses (out on) its “distinctive felt qualitative character” (Sheets-Johnstone, 2011: 122) and inherent, complex, and nuanced “spatio-temporal-energetic” (2011: 432) dynamic structures. We must not forget that *The Dot and the Line* is accompanied by a human narrator, a love story, and an expressive musical score. Granted, animation techniques, and underlying physics engines have much advanced since then, but a character’s affective potential still relies on dramaturgically framing and timing it inside the bounds of the screen; e.g., in John Lasseter’s *Luxo Jr.* (1986), “it was very important that the audience was looking in the right place at the right time” (Lasseter, 2001).

Physical puppeteering techniques, in contrast, connect the puppeteered to the human puppeteer in a shared physical space, although this connection is if often described as a form of manipulation—manipulating the puppet or its movable parts (see Piris, 2014), rather than extending into it/them. In our PBM studies, dancers sometimes chose to position themselves outside the PBM costume to, essentially, puppeteer it (opposed to inhabiting and moving *with* it). According to their own accounts, this felt more like attempting to control the costume, clutching it with their hands, arms, or legs and using their visual sense to explore movement patterns.^35^ The entangled approach, in contrast, requires negotiation rather than control, which, we found, opens up a rich, ample landscape of affordances (see Rietveld and Kiverstein, 2014) from within. Drawing on my insights from our embodied design process and audience observations, I argue that the “spatio-temporal-energetic” (Sheets-Johnstone, 2011: 432) richness of movement qualities offers more than cues on possibilities. It *generates* possibilities by unfolding relational affordances that give rise to intracorporeal meaning-making, which is core to social understanding (Fuchs and Koch, 2014). The latter “is not an inner modeling in a detached observer,” but rather the “other’s body extends onto my own, and my own extends onto the other” (Fuchs and Koch, 2014: 6). Scaffolding the robot’s learning by transforming human movement patterns in relation to the robot’s unique embodiment, I argued, gives rise to a *bodying-thinging* in which a 75 × 75 × 75 cm cube-in-motion extends toward my body, and my own extends toward this other “bodying forth” (see Manning and Massumi, 2014).

Watching participants, whether as unsuspecting audience members or in studies, encounter our Cube Performer for the first time, one of the most common reactions, is surprise (see also Levillain and Zibetti, 2017). I am often reminded of Haraway’s sometimes jerky, sometimes flowing dance that is embodied communication, where the dynamics unfold in unpredictable configurations and participants find themselves, alternating, in moments of harmony or “painfully out of synch” (2008: 26) with the cube. Some people reach out and rhythmically coordinate with its movements with their hand gently touching one of its sides. Others prefer to step back and observe it for a while, usually circulating around it. Quite often, something makes them smile at the artifact or elicits a giggle. Participants also often crouch to match the Cube Performer’s height or, more rarely, even take turns with the cube on their hands and knees whilst trying to keep up with its, at times, quick accelerations. In general, people either leave within 2 min or engage with it for more than 5 min, sometimes significantly longer. In the latter encounters, we found the following characteristics: interactors are 1) occupied with probing how the robot “works” and/or how they are being sensed, 2) engaged in an interplay of following the robot’s movements, even tilting with it, etc., and attempting to elicit responses from it by moving in unexpected ways, or 3) inquisitive regarding its workings at first and then seemingly begin to settle and move in accordance with the robot (similar to 2). Interactors we talked to referred to the Cube Performer in surprisingly affective terms, using words like “gentle,” “timid,” “aggressive,” “competitive,” “cheeky,” or “playful” to describe the ways in which it moves. Although the robot still lacked improvisational skills at the time of these public encounters, many participants related its movements to their own. Talking about having felt observed by the object, one participant said, “I know it’s very connected … it’s obvious that it does what it does because I’m here.” To another, the cube came “across as playful with an ‘honest curiosity’.” Another commented, “I was sort of surprised about how intimate it felt … I felt quite tender toward it.”

### Design Challenges

The bodily, kinaesthetic immersion, which is at the core of our PBM and RBM mapping instruments, renders them very specific and is not feasible or practical in any design approach or for any potential robotic form. My account of designing with these embodied interfaces, however, offers the more readily transferable insights that careful attention to movement qualities in relation to a robot’s specific embodiment can contribute to 1) situate abstract robots and 2) generating meanings as part of the interaction process. My notion of *designing with* suggests that we place more attention onto the embodied, imaginary and material meaning-making encounters afforded by the design process itself and how they shape the robot’s social abilities.

Even if we have the opportunity to work with professional movement experts and wearable costumes or prosthesis-like attachments (see Gemeinboeck and Saunders, 2017) or any other types of mock-ups that performers can be creative with (see Hoffman and Ju, 2014; Sirkin et al., 2016), the delay between this, often, improvisational process and the technical development is significant. That is, the various technical design, prototyping, and machine learning stages involved in robot-making cannot keep up with the pace in which embodied knowledge and questions are produced in the experimental process with the performers. This inevitably slows down and, at stages, compromises the necessary and rich knowledge transfer and feedback loops between experimental and technical processes and the embodied insights they offer.

Studying the social, relational and performative effects of agential enactment as they unfold in the interaction dynamics is very challenging. As we aim to give ample space to meanings and relations emerging and being negotiated in the encounter, there are no specific tasks or predefined social capacities against which we can measure how well the robot or a human-robot-coupling performs. Observed relationships often do not discriminate between relations that emerge from within the encounter and ones that arise (only) in the eye of the observer. While we found that participants asked about their experience often were able to identify salient moments that triggered something, they may find it challenging to further articulate what happened in these moments of dis/connectedness, particularly in the first encounter. Also, as we look at meaning-making unique to each encounter, results are not as decisive and comparable as in more typical study setups. These challenges, however, are not only reserved for studying performative relations with machinelike artifacts but tend to arise when studying the complex dynamics of social interactions as situated couplings (see Bombari et al., 2015; De Jaegher et al., 2017).

Tightly interlinked with the above is the challenge that we can no longer control or predict what happens in the encounter nor whether or how meaningful social relations emerge. From a traditional engineering viewpoint, this may sound like something that only an artistic project can afford or even desires. Indeed, MML deliberately pursues design strategies that render our intra-actions with machines more emergent, open, and potentially ambiguous and definitely irreproducible (see also Levillain and Zibetti, 2017). I recognize that this is not a feasible or desirable strategy for any robot design or human-robot interaction scenario. But, I also strongly believe that we can only advance our knowledge about possible, meaningful relations with machines and what a robot *could* be, if we invest in more diverse and differentiated approaches into *designing with* a machine’s social potential.

## Summary

My relational-performative framework understands aesthetics as central to our embodied meaning-making in human-machine relationships. Agency and, with it, sociality are enacted in the situated dynamics of the interaction itself, which moves the design focus into the middle of the encounter. Drawing on a performative new materialist account and embodied, enactive meaning-making, possible human-robot relationships are not a matter of design but rather are to be negotiated and *designed with* to give ample space to the interactional situation and the transformative potential of its social dynamics. Integral to this transformational process is the potential for *bodying-thinging*, where relations are not the product of meaning-making experiences but instead constitute meanings and experiences and, with them, subjects and objects. *Bodying-thinging* foregrounds both human and nonhuman capacities to extend toward and across boundaries and, doing so, destabilizes and potentially collapses binary opposites (Fischer-Lichte, 2008). A relational-performative aesthetics thus counters fixed, superficial esthetic mappings in human-robot interaction design and demystifies the figure of the robot as an independent, autonomous agent.

My notion of *designing with* proposes that we find ourselves in the midst of “the encounter” from the early stage of the design process. This is where we begin to manifest boundaries that shape meaning-making and the potential for emergence, transformation, and connections to arise from intra-bodily resonances (*bodying-thinging*). Discussing creative motion-centric approaches that put aesthetics to work to reimagine human-machine boundaries, I explored different perspectives on agency and how it constitutes the robot/machine as performer. My collaborative Machine Movement Lab project opens up an intimate link to performance-based inquiries into the relational enactment of human-robot encounters, based on an aesthetics arising from difference in relation. The Performative Body Mapping (PBM) methodology harnesses dancers’ tactile-kinaesthetic expertize and the sociocultural dimensions of (human) movement qualities to socially situate an abstract robotic artifact and bootstrap its machine learning. Relational Body Mapping (RBM) extends the PBM costume to serve as an instrument for sensorial mapping between different human and nonhuman perceptual worlds. MML’s overarching aim is to open up new pathways for robot design by focusing on the coupling of human-machine and “giving space” to the enactment of relations and the emergence of meanings in the encounter.

According to Suchman, reconceptualizing how we conceive of “the human, the technological, and the relations between them [has] implications for everyday practices of technology design” (2007b: 139). Reconceptualizing our relations with robots and how this implicates and transforms our design practices has been the main focus of this article. How such alternative practices can affect new kinds of relationships with robots will require significant investment into studying differentiated and diverse design approaches as well as involving potential users from the early stage of the design process. Creative practices expand technology design not only by bringing different cultural and esthetic questions to human-robot interaction research but also by engaging the publics in the important question of how machines *could* socially participate in our society.

## Data Availability

The original contributions presented in the study are included in the articlel; further inquiries can be directed to the corresponding author.

## Footnotes

1 By situated (adj.) or situatedness (n.), I refer to something, e.g., an interaction, occurring in a particular situation, and, importantly, the particulars of the situation playing a key role in how the interaction unfolds. Situated here thus means being bound to a particular physical, sociocultural, and historical context. Both, situatedness and embodiment have increasingly become important concepts in cognitive science (see Suchman, 1987; Varela, Thompson and Rosch, 1991; Pfeifer and Scheier, 1999), rejecting traditional cognitive-scientific notions of internal representation and computation in favor of studying the fundamental role of bodily mechanisms and the environment, including interactions with other agents, artifacts, etc. (Ziemke, 2002).

2 The concept of performativity (adj.: performative) was developed by J. L. Austin to study speech acts and has since then been drawn on and extended by numerous theorists, most notably J. Derrida, J. Butler, D. Haraway, and K. Barad. My esthetic approach draws on Barad’s (2003) and Suchman's (2007) account of performativity, albeit the scope of this article does not reach into the important discursive dimensions of gender and labor within the context of social robot design. In particular, my relational-performative approach aims (1) to shift the focus from a representationalist to a performative understanding of agency in human-robot interaction, and (2) to look at human-robot interaction as a practice involved with the making and configuring of boundaries between humans and nonhumans or subjects and objects. Importantly, the adjective “performative,” as used here, does *not* refer to the nature of dramatic or artistic performance.

3 Co-directed with Rob Saunders.

4 Jochum et al. state that “[w]here classical engineering favors precision, artists are trained to look for creative solutions by exploring ambiguity and uncertainty” and “transforming design and technological constraints into advantages” (2017: 374).

5 This argument not only draws on my own collaborative practice but also ethnographic studies of interactions in social robotics laboratories that demonstrate the importance of situational dynamics and the role they play in human involvement in a robot achieving social agency (Alač 2011; Alač, 2015; see also Suchman, 2007a).

6 My brief argument here draws on a much deeper and wider historical and political discourse, e.g., see Suchman, 2007b; Suchman, 2007a; Suchman, 2011; Haraway, 1989; Haraway, 2003; Barad, 2007; Barad, 2003; Hayles, 1999; Latour, 1993; Penny, 2017; Gemeinboeck, 2019, to just name a few.

7 The costumes were built with lightweight fluted plastic or plywood panels and plastic tubes, see Figure 2. A detailed account of this early form-finding stage, selection criteria and movement experiments can be found in (Gemeinboeck and Saunders, 2017).

8 Theater and performance have a history using costumes to “transform” performers’ performance. For example, for his 1993 production of *Tristan and Isolde*, Heiner Mueller asked Yohji Yamamoto to design costumes for the singers “that would impede on the movement they are used to” (Suschke 2003: 205). Rather than interfering with the dancer’s movement, however, we are looking for a productive intermeshing (see *Designing with Bodying‐Thinging*).

9 Often discussed within the context of demonstration learning in HRI, the correspondence problem refers to the challenge of mapping between two very different embodiments (Dautenhahn et al., 2003).

10 I refer here to the work of our collaborator, choreographer Tess De Quincey, and her mental image of the “nervous body,” developed as part of her *BodyWeather* practice (see https://dequinceyco.net, accessed on 17 June 2020). Sourced from a video recording of a PBM movement study, 26 March 2016 (unpublished). For more discussion of mental imagery in dance see also Foster, 2000.

11 We took the opportunity of turning one of our iterative prototyping stages into building a complete, second prototype to have two robotic artifacts to work with, e.g., for studying the performative potential of machine-machine couplings or incorporating them in performance settings with human performers (see *Dancing with the Nonhuman*). Descriptions of our first design of a robotic artifact, the *Cube Performer*, its mechanical design and early machine learning stages can be found in (Gemeinboeck and Saunders, 2017; Gemeinboeck and Saunders, 2018; Saunders and Gemeinboeck, 2018).

12 We conducted two participatory studies: the first three-day study involved 48 participants and took place as part of the exhibition *RePair* at the *Big Anxiety Festival*, Sydney, November 2017; the more detailed follow-up study, in February 2019, involved ten participants as part of a two-day open lab, both situated in the same performance space at the UNSW Art and Design campus. Details on study design and discussion of results can be found in (Gemeinboeck and Saunders, 2018; Gemeinboeck and Saunders, 2019). In this article, I take an anecdotal approach to reporting participants’ feedback to complement my conceptual/theoretical argument with material-experiential accounts.

13 Suchman reminds us that mutualities are not necessarily symmetries, suggesting “that persons and artifacts do not constitute each other *in the same way*” (2007: 269). Contrasting representational approaches to human-robot interaction design, this relational-performative approach embraces the asymmetries between persons and artifacts.

14 Discussing her notion of coping, Noland talks about co-construction, where dances and bodies are “performatively produced in dialogue with external devices” (2009: 3).

15 The cube costumes consist of a lightweight aluminum frame, 75 × 75 × 75cm, with either five plywood or three plywood and two Plexiglas faces (see Figure 5).

16 Based on an unpublished interview with Audrey Rochette on 3 July 2019, University of Applied Arts Vienna.

17 Dancers who inhabited the PBM costume (nine in total) have stated that their proprioceptive sense increasingly extends to the performer-cube entanglement (based on conversations with collaborating performers captured in video recordings of movement studies, on 31 January 2017, 15 March 2018, 24 January 2019, 3 July 2019 (unpublished).

18 I refer here to our participatory study in 2019, involving ten participants as part of a two-day open lab at a performance space at the UNSW Art and Design campus, discussed in more detail in (Gemeinboeck and Saunders, 2019).

19 This is not a figure of speech. As our kinaesthetic experiences are grounded in embodiment (Johnson, 2007; Sheets-Johnstone, 2012; Lindblom, 2015), our thoughts and feelings are equally grounded in our bodily interaction with other bodies, things and the environment (see Meier et al., 2012; Fuchs, 2016). They manifest in embodied ways in what Froese and Fuchs have termed “intra-bodily resonance” (Froese and Fuchs, 2012: 212) and express themselves to others, interpreted, in turn, via intra-bodily resonance. *Bodying-thinging* aligns Froese and Fuchs’ intra-bodily resonance with Despret’s ethological concept of “embodied empathy” (2013).

20 Based on their cognitive psychology studies, Levillain and Zibetti argue that objects’ distinct behavior produces transformations that trigger “the same kind of attributions that would be activated by the motion of a living being” (2017: 13).

21 It may be worth clarifying that my argument for our encounter beginning with the early stages of design does not require specific design techniques, such as PBM or bodystorming. The latter is a technique from experience design and differs from PBM in that it focuses on looking at a (not yet existing) product from a user’s perspective, e.g., by simulating the environment of use or by “employing ‘actors’ and ‘props’” to act out possible ways of use (Schleicher et al 2010). While my notion of *designing with* emphasizes embodied meaning-making, it does not refer to designers projecting themselves into the user’s position (although this is always a desirable strategy when we design for others). Speaking to the beliefs and intents that we bring to the design process (e.g., by shifting from a representational to a relational approach), my argument is that, from a relational-performative perspective, the embodied, material meaning-making of our design processes already affords us and constitutes a situation of encounter.

22 We also built a second costume in the form of a tetrahedron and studied and probed into its kinetic capabilities (see Figure 2). Built of elastically connected pipes, a serendipitous accident soon turned the simple tetrahedron shape into a 5-jointed, broken tetrahedron This is still a robot in waiting (see Gemeinboeck and Saunders, 2017).

23 It is this omnidirectional, regular geometry that usually lends specific functionalities to cube shaped robots, e.g. in reconfigurable modular robot design (see Brunete et al., 2017).

24 Sadly, I have not had the opportunity to encounter Petit Mal myself.

25 My earlier artwork, *Accomplice* (2012–14), was similarly conceived as a robotic anti-spectacle: embedding four robots into the architectural fabric of the gallery; they are mainly present through the material traces of their workings, and audiences can only catch glimpses once they cracked open the wall as they track their relentless knocking sounds (Gemeinboeck and Saunders, 2016a). Accomplice by Petra Gemeinboeck and Rob Saunders is a machine installation that generates its own performance space over time: https://vimeo.com/101790975 [Accessed on 21 April 2020].

26
*RePair* at *The Big Anxiety Festival,* Sydney, 2017, and the *Performing Arts* and *Games Festival 2018*, United Kingdom.

27 UNSW Art and Design, February 2019. Details can be found in (Gemeinboeck and Saunders, 2019).

28 It is important to note that favoring behavior over form is by no means unique to these three works. Already in 1997, Kac (1997) identified artists “giving precedence to behavior over form” as a principle of robotic art. Penny also stresses this point in (2017).

29 Dancing with the Nonhuman is a three-year project and, at the time of writing, is nearing the end of its first year.

30 Other recent examples of collaborations between performance and robotics that develop performance-led methodologies include St-Onge et al. (2019) exploration of robotic swarms and how motion-based expressivity can convey information, and Jochum et al. (2016) employing applied theater as a platform for studying human-robot interaction (see also Relevant Positions and Practices). The performance project *Grace State Machines* (2007) by Bill Vorn, Emma Howes and Jonathan Villeneuve explores questions of kinaesthesis and perception in a dialogue between machine performers and a dancer: https://billvorn.concordia.ca/robography/GraceState.html (accessed on 12 October 2020).

31 Sensors include accelerometers, gyroscopes, magnetometers, depth cameras and a LiDAR.

32 Developed in collaboration with co-investigator and choreographer Marie-Claude Poulin; see: http://www.konditionpluriel.org (accessed on 20 October 2020).

33 For example, in relation to vulnerable populations (see Šabanović, 2010; Turkle, 2011; Castañeda and Suchman 2014; Jones, 2018).

34 For a more detailed discussion of animation techniques in relation to the affordances provided by performance-based techniques; see Gemeinboeck and Saunders (2106b).

35 Based on my conversations with Tess De Quincey and Kirsten Packham. Video recording, 31 January 2017, Sydney (unpublished). See also Gemeinboeck and Saunders (2017).
